# Design, synthesis and biological evaluation of antimalarial activity of new derivatives of 2,4,6-*s*-triazine

**DOI:** 10.1186/s13065-017-0362-5

**Published:** 2017-12-19

**Authors:** Mallika Pathak, Himanshu Ojha, Anjani K. Tiwari, Deepti Sharma, Manisha Saini, Rita Kakkar

**Affiliations:** 1 Department of Chemistry, Miranda House, University of Delhi, Delhi, 110007 India; 2 Department of Chemistry, University of Delhi, Delhi, 110007 India; 3 Division of CBRN Defence, Institute of Nuclear Medicine and Allied Sciences, DRDO, Timarpur, Delhi, 110054 India

**Keywords:** Antimalarial, DHFR inhibitors, Molecular docking, *s*-Triazine, 3D7 strain

## Abstract

Dihydrofolate reductase (DHFR) is an important enzyme for de novo synthesis of nucleotides in *Plasmodium falciparum* and it is essential for cell proliferation. DHFR is a well known antimalarial target for drugs like cycloguanil and pyrimethamine which target its inhibition for their pharmacological actions. However, the clinical efficacies of these antimalarial drugs have been compromising due to multiple mutations occurring in DHFR that lead to drug resistance. In this background, we have designed 22 *s* ***-***triazine compounds using the best five parameters based 3D-QSAR model built by using genetic function approximation. In-silico designed compounds were further filtered to 6 compounds based upon their ADME properties, docking studies and predicted minimum inhibitory concentrations (MIC). Out of 6 compounds, 3 compounds were synthesized in good yield over 95% and characterized using IR, ^1^HNMR, ^13^CNMR and mass spectroscopic techniques. Parasitemia inhibition assay was used to evaluate the antimalarial activity of *s* ***-***triazine compounds against 3D7 strain of *P. falciparum.* All the three compounds (**7**, **13** and **18**) showed 30 times higher potency than cycloguanil (standard drug). It was observed that compound 18 was the most active while the compound **13** was the least active. On the closer inspection of physicochemical properties and SAR, it was observed that the presence of electron donating groups, number of hydrogen bond formation, lipophilicity of ligands and coulson charge of nitrogen atom present in the triazine ring enhances the DHFR inhibition significantly. This study will contribute to further endeavours of more potent DHFR inhibitors.

**Electronic supplementary material:**

The online version of this article (10.1186/s13065-017-0362-5) contains supplementary material, which is available to authorized users.

## Introduction

Malaria is a protozoan disease caused by *Plasmodium* genus. According to WHO report entitled “World malaria report” (2015), 15 countries reported 80% of cases and 78% of deaths due to malaria in 2015 [1]. Malaria persists to be one of the critical public health problems in India. Around 1.13 million confirmed cases and 287 deaths were reported in 2015 by the National Vector Borne Disease Control Programme (NVBDCP), out of which 67.1% was due to *Plasmodium falciparum* [2]. Odisha, Jharkhand, Chhattisgarh, Madhya Pradesh, Karnataka and north-eastern states except Sikkim, Maharashtra and Rajasthan are high endemic areas in India.

Antifolate antimalarial drugs such as pyrimethamine and cycloguanil have been used in prevention and treatment of malaria. It is well known that folate metabolism is one of the most studied biochemical pathways of the parasite. Folate metabolism is a critical process being targeted to stop the proliferation of the parasite. The antimalarial activity of therapeutic agents that interfere with folate metabolism has been recognized since long. Two categories of antifolate antimalarial drugs were distinguished by their respective mechanisms of action. In the first category, the sulphonamides and sulphones are chemical analogues of *p*-amino benzoic acid (PABA), an essential precursor for the de novo synthesis of folic acid. The second category includes a variety of drugs that inhibit dihydrofolate reductase (DHFR), the enzyme responsible for converting dihydrofolate to the biologically active tetrahydrofolate cofactor [3].

During the earlier trials of chloroguanide as an antimalarial drug on monkeys, rabbits and humans, triazine compounds were identified and isolated. Among these compounds 2-amino-4-(*p*-chloro-anilino)-6,6-dimethyl-5,6-dihydro-1,3,5-triazine and *p*-chlorophenylbiguanide were isolated from the urine of monkeys treated with chloroguanide [4]. But both the compounds were found to be inactive against *Plasmodium falciparum.* However, an isomer of 2-amino-4-(*p*-chloro-anilino)-6,6-dimethyl-5,6-dihydro-1,3,5-triazine,2,4-diamino-5-(*p*-chlorophenyl)-6,6-dimethyl-5,6-dihydro-1,3,5-triazine, isolated from the urine of rabbits [5] and humans [6] were found to be highly active. A large number of dihydrotriazines have been synthesized and many of them showed antimalarial activity [7, 8]. Further, several works have been reported to study the correlation between the structure and the antimalarial activity of triazine compounds. Based on these relationships, several triazine compounds have been synthesized and biologically evaluated for biochemical targets such as polyamine metabolism [9] and DHFR inhibition [10, 11, 12].

The synthesis of *s*-triazines and their pharmacological applications are well documented [13, 14, 15, 16]. Some *s*-triazine derivatives are reported to possess remarkable antitubercular [17], antimicrobial [18], antibacterial [19] and herbicidal activities [20]. Besides it, *s*-triazine compounds were found to be active as antitumorigenic agents, in chemotherapeutical preparations, active against viruses, protozoa, helminths, pharmacologically effective to treat cardiovascular, neuropsychotic disorders, or inflammatory processes, diuretics, antidiabetic agents, etc. [21].

According to the literature [22, 23] *s*-triazine compounds fall into the second category that inhibits *Plasmodium falciparum*-DHFR. DHFR has received considerable attention as it is the target of cycloguanil (a triazine based antimalarial drug) and other antifolates. DHFR is used for prophylaxis and the treatment of *Plasmodium falciparum* infection [24]. The exponential increase in the emergence of antifolate resistance in *Plasmodium falciparum* has unfortunately compromised the clinical use of the currently used drugs and therefore highlights the urgent need for new effective antifolate antimalarials [25, 26].

During the last two decades, there has been tremendous progress in computational chemistry and Computer Aided Drug Design (CADD). CADD has played a major role in screening of new chemical entities. Under ligand based lead compounds optimization, QSAR study of the bioactive compounds plays a useful role for screening of new potential lead compounds. Therefore, the design of novel chemical entities which can affect selectively the parasite folate metabolism, may lead to discovery of better antimalarial drugs. In our previous reported study we had prepared and discussed 3D QSAR models using Genetic Function Approximation (GFA) method by employing data set of minimum inhibitory concentration (MIC) values of synthetic *s*-triazine compounds tested for DHFR inhibition against cycloguanil resistant strain of *Plasmodium falciparum* [27]. Using QSAR model no 1 (best model), a number of *s*-triazine compounds were designed by modifying the attached side chains to the carbon atoms of the triazine ring of the parent compound (Table 1).

**Table 1:** Structural features of the designed inhibitors and predicted pMIC values

Designed inhibitor	R group	Predicted log1/*MIC*	Designed inhibitor	R group	Predicted p*MIC*
**1**		− 4.47	**12**		− 3.46
**2**		− 3.84	**13**		− 0.90
**3**		− 3.19	**14**		− 2.93
**4**		− 3.41	**15**		− 1.85
**5**		− 2.62	**16**		− 2.29
**6**		− 2.78	**17**		− 3.23
**7**		0.65	**18**		0.44
**8**		0.19	**19**		− 2.2
**9**		− 0.13	**20**		− 3.88
**10**		0.58	**21**		− 4.05
**11**		− 2.76	**22**		− 6.8

Under the present study new *s*-triazine compounds were designed and their MIC values were predicted using same QSAR model. The designed compounds were also evaluated for ADME properties and docking score. Based upon these parameters 6 *s*-triazine compounds were selected for synthesis. Out of 6 compounds, 3 compounds were synthesized with yield percentage above 95%. The compounds were characterized using elemental analysis, IR, mass, ^1^HNMR and ^13^CNMR experimental techniques. The synthesized compounds were tested against the 3D7 strain of *Plasmodium falciparum* Rieckmann microassay [12, 16]. It was observed that all synthesized compounds possessed 30 times higher activity than the standard cycloguanil antimalarial drug.

## Materials and methods

### Chemicals and techniques

All chemicals used in the present study are of analytical grade purchased from Sigma Aldrich and Merck Chemical Company. All the solvents were used after distillation. All the synthesized compounds have been characterized from their analytical, physical and spectral (IR, ^1^HNMR, ^13^C-NMR) data. Infrared spectra (IR) spectra were recorded in KBr discs on an FT-IR Perkin-Elmer spectrum BX spectrophotometer. ESI–MS spectra were obtained using a VG Biotech Quatrro mass spectrometer equipped with an electrospray ionization source in the mass range of *m/z* 100 to *m/z* 1000. ^1^H-NMR and ^13^C-NMR spectra were recorded on a Bruker NMR instrument 400 MHz and 100 MHz, respectively using CDCl_3_ and DMSO-d_6_ as solvents. Elemental analysis was performed on the elemental analyzer Gmbh variable system. All compounds gave satisfactory analytical results.

### ADME screening

QikProp program from Schrödinger Mastero 9.7 [28] was employed to assess the absorption, distribution, metabolism, and excretion (ADME) properties of the compounds. QikProp predicts both pharmaceutically significant descriptors and physically relevant properties. The program was processed in the normal mode, and 44 properties were predicted for the 22 *s*-triazine compounds. These predicted properties consist of principal descriptors and physicochemical properties with a detailed analysis of the octanol/water partition coefficient (QlogPo/w), octanol/gas partition coefficient (QlogPoct), water/gas partition coefficient (QlogPw), polarizability in cubic A^0^ (QPolrz), % human absorption in the intestines (QP%), brain/blood partition coefficient (QPlogBB), IC_50_ value of HERG K^+^ blockage channels (logHERG), skin permeability (QPlogKp), binding to human serum albumin (QPlogKhsa), apparent Caco-2 cell permeability in mm/s (QPPCaco), and apparent MDCK cell permeability in mm/s (QPPMDCK). Caco-2 cell line is good model for the gut-blood barrier, while MDCK cell line is considered a good model for the blood–brain barrier. Besides, QikProp evaluates the acceptability of the compounds based on Lipinski’s rule of five [29], which is essential for rational drug design. Low permeability and/or poor absorption for compounds results when a compound violates one or more than one Lipinski’s rule of five (i.e. more than 5 hydrogen donors, the molecular weight is over 500, the logP is over 5 and the sum of N’s and O’s is over 10).

### Chemistry

#### General procedure for the synthesis of compounds (**7**, **13** and **18**)

The 2,4,6-trisubstituted-1,3,5-triazine compounds were synthesized by refluxing 2,4,6-trichloro-1,3,5-triazine (cyanuric chloride) with different nucleophiles (R). The mono-substituted triazine (4,6-dichloro-*N*-(4-nitrophenyl)-1,3,5-triazin-2-amine) was synthesized by refluxing cyanuric chloride with *p*-nitroaniline in the presence of potassium carbonate in tetrahydrofuran (THF).

##### *N*-2-(4-Nitrophenyl)-*N*-4,*N*-6-bis[3-(pyridin-2-*yl*)propyl]-1,3,5-triazine-2,4,6-triamine (**7**)

Yellow (solid). Yield 96%. Mp: 144–1461 °C; IR (KBr, υ_max_ in cm^−1^): 3412 (N–H, str.); 1643 (C=N, str.); 1537, 1372 (NO_2_, str.); ^1^H-NMR (400 MHz, CDCl_3_): 8.1–6.3 (m, 12H, Ar–H); 4.5 (m, 4H, CH_2_); 3.5 (t, 4H, CH_2_); 2.8 (t, 4H, CH_2_); 5.6 (s, 1H, NH); ^13^C-NMR (100 MHz, CDCl_3_) δ, ppm: 172.8, 169.5, 164.3, 154.2, 145.3, 141.9, 137.6, 131.2, 127.2, 113, 100.9, 79.0, 57.9, 54.6, 39.9; Anal. Calcd. for C_25_H_27_N_9_O_2_ C: 61.62; H: 5.25; N: 24.71; found: C: 61.50; H: 5.91; N: 25.86. Mass spectrum (ESI) (M + H)^+^ = 486.6

##### 3-[4-(3-Hydroxyphenylamino)-6-(4-nitrophenylamino)-1,3,5-triazin-2-ylamino]phenol (**13**)

Black (solid). Yield 98%. Mp: 180–182 °C; IR (KBr, υ_max_ in cm^−1^): 3409 (OH, str.); 1631 (C=N, str.); 1591, 1326 (NO_2_, str.); 1180 (C–O, str.); ^1^H-NMR (400 MHz, DMSO-*d*_6_): 9.1–7.4 (m, 12H, Ar–H); 5.9 (s, 3H, NH); ^13^C NMR (100 MHz, DMSO-*d*_6_) δ ppm: 169, 167.4, 152, 135.4, 131.7, 126.7, 123, 120.9, 119.3, 117.3; Anal. Calcd. for C_21_H_17_N_7_O_4_ C: 58.47; H: 3.97; N: 22.73; found: C: 58.53; H: 4.01; N: 22.64. Mass spectrum (ESI) (M + H)^+^ = 432.1

##### 4,6-bis(4-Ethylpiperazin-1-yl)-*N*-(4-nitrophenyl)-1,3,5-triazin-2-amine (**18**)

White (solid). Yield 97.5%. Mp: 160–161 °C; IR (KBr, υ_max_ in cm^−1^) 3293 (N–H, str.); 1599, 1660 (C=N, str.); 1541, 1317 (NO_2_, str.); ^1^H-NMR (400 MHz, CDCl_3_): 7.6–7.1 (m, 4H, Ar–H); 3.9 (q, 4H, CH_2_); 3.5 (s, 1H, NH); 3.28 (t, 4H, CH_2_); 2.9 (t, 6H, CH_3_); ^13^C NMR (100 MHz, CDCl_3_) δ ppm: 172.1, 155.3, 141.8, 135.7, 130.4, 114.3, 82, 77.3, 55.6, 43.5, 37.6; Anal. Calcd. for C_21_H_31_N_9_O_2_ C: 57.13; H: 7.08; N: 28.55; found: C: 57.07; H: 7.11; N: 28.52. Mass spectrum (ESI) (M + H)^+^ = 442.9

### Pharmacology

#### Plasmodium parasite culture

Stock culture of malaria parasite *Plasmodium falciparum* 3D7 strain was continuously maintained in vitro using the candle-jar method [30]. The *Plasmodium falciparum* 3D7 strain was maintained on B^+^ human red blood cells. The aqueous culture media (960 mL) consisted of 10.4 g of RPMI-1640 with 40 mg of gentamicin and 5.94 g of HEPES buffer. The culture medium was reconstituted just before use by pouring sterile 5% sodium bicarbonate in ratio of 1:24 and the culture was further supplemented with 10% Bovineserum. The parasitemia culture was maintained in between 1 and 5% and routinely sub-culturing was performed on every fourth day. The hematocrit was maintained initially at 7%.

#### Plasmodium dilutions preparation

Each compound was dissolved separately in DMSO to obtain stock solutions of 1 mg/mL concentration. The graded concentration of each compound used was as follows: 10, 5, 2, 1, and 0.1 µg/mL. The working solutions of the desired concentration were prepared freshly by diluting the stock solutions of compounds. The final concentration of DMSO used in the culture media did not affect the parasite growth.

#### Inhibitory concentration assay

The minimum inhibitory concentrations of each compound were determined in vitro using a dose–response assay in 24-well tissue culture plates in triplicates. Synchronous parasites were prepared [31] to obtain parasitized cells harbouring only the ring stage and challenged with a graded concentration ranging from 0.1 to 10 µg/mL of the drug solution for 48 h at 37 °C by the candle-jar method [30]. The medium was changed routinely after 24 h in each of wells (with or without the drug). Thin smear with Giemsa-staining were prepared and analyzed to determine the percentage inhibition of parasitemia vis-a-vis the control.

#### Plasmodium slide preparation

The 96-well plates were taken out from the candle jar and the material from each well was transferred into the corresponding well labelled 1.5-mL microcentrifuge tube. After vortexing, the supernatant was pipetted out and the pellet was further spread thoroughly on a slide to prepare a thin blood smear slide for each well. Subsequently the smeared slides were air-dried, fixed with methanol and stained with Giemsa dye for 40 min. After staining, the excess dye was removed by washing the slides in running tap water and finally slides were again air-dried. The stained slides were examined in random adjacent microscopic fields to count the number of parasites equivalent to approximately 3000 erythrocytes at 100× magnification.

## Results and discussion

### Design of new inhibitors

In the QSAR model, the following properties appear in the top-most equations: *χ*(3) cluster, *κ*(1), Wiener index, Coulson charge on N_3_, Electrostatic charge on C_2_, Dipole moment (x), Total dipole, Octupole moment and Total energy. This list indicates that structural (topological) as well as electronic factors contribute to the activity or inactivity of a given compound. However, we require a deeper introspection of the actual quantitative effect of these parameters on the activity value. Deciphering the information available from a QSAR model needs the study of coefficients of these properties as they appear in the top equations.

The most powerful factor here is the charge on the nitrogen atom of the triazine ring, sandwiched between the two side chains. This indicates that the electron density at the triazine group should not decrease. So it was rational to attach electron donating atoms in these side chains.

χ(3) cluster contributes negatively towards activity. It leads us to keep less clustering in the side chains. κ(1) has a positive coefficient, though of a comparatively lower value. It signifies that contacts of first degree between atoms are beneficial in improving the activity or we can say that branching is not a favourable trait. Clustering could result in bad grades. Very long chains are also not recommended as elongation of the side chain has no major effect on the electronic contribution towards activity. These points motivated us to choose simple 2–3 carbon atom chains to be introduced near the triazine moiety.

Considering the factors described above, a series of R groups were attached to the triazine ring. Structural features of the compounds so obtained are given in Table 1, along with the predicted p*MIC* values, based on the first QSAR equation, for the corresponding derivatives.

1 $${\text{Y}} = 0. 2 3 8 7\left( 1\right) - 2. 2 70 4\left( 3\right) - 0. 30 1 4\upmu_{{\rm T}} - 0. 2 20 7\upmu_{\text{x}} - 00. 7 9 3 5 {\text{q}}_{\text{c}} - 3 7. 4 6 9 5$$

where, κ(1) is the shape descriptor, χ(3) is the molecular connectivity indices, µ_T_ is the total dipole moment, µ_x_ is the dipole moment in the X direction and q_c_ is the coulson charge on nitrogen atom.

It can be seen that substitution of electron donating functional groups at various positions lead to an increase in the activity of the derivatives. It is clear that attachment of an electron acceptor decreases the predicted value. An alcoholic group reduces p*MIC* to − 4.47 (compound **1**). Replacement of –OH with –NH_2_ improves the activity to a small extent (compound **2**). Therefore various kinds of groups, such as phenyl, heterocyclic aromatic and aliphatic 5–6 member rings, and small aliphatic chains, were taken and the –NH_2_ group was added at different positions on the chain and at rings so as to get higher p*MIC* values (example—compounds **3**, **9**). Addition of methylamine and ethylamine proved to be better than amine (e.g. compare the pairs compounds **5** and **6**; **15** and **16**). Elongation of chain length also results in slightly better activity. An additional methyl group in the chain causes a slight increment in the biological activity. This can be seen as we move from compound **2**–**3**. However, clustering and branching of any kind is not at all beneficial. Whenever an isopropyl or isobutyl group is added instead of an ethyl or methyl group, the activity for the resulting compound decreases (as in case of **4**, **11** and **12**). In this course of action, we obtained new compounds which had better value than the existing compounds used in the QSAR study. Based on the overall analysis we can conclude that the compounds **7**, **8**, **9**, **10**, **13** and **18** (with p*MIC*: 0.65, 0.19, − 0.13, 0.58, − 0.90 and 0.44, respectively) are the most potent derivatives that could prove to be better drugs than the existing ones.

### ADME analysis and molecular docking

In ADME screening, 44 parameters were calculated, which included molecular descriptors and pharmaceutically relevant properties like the partition coefficient (logPo/w) and water solubility (logS), critical for estimation of absorption and distribution of drugs within the body, the blood brain barrier permeability (logBB) which is prerequisite for the entry of drugs to the brain, (log Kp) predicted skin permeability, (logKhsa) prediction of binding to human serum albumin, (P_caco_) model for the gut-blood barrier, percentage of human oral absorption and Lipinski’s rule of five was considered a parameter to screen the best candidates out of 22 compounds.

It was observed that out of 22 compounds only 6 lead compounds were found not violating any of ADME property while the rest of 16 designed compounds have violated few important ADME properties like P_caco_, which is predicted apparent Caco-2 cell permeability in nm/s and are good model for gut-blood barrier, logKhsa that is the prediction of binding of ligand to the human serum albumin which in turn influence the biodistribution of drug in the blood and Lipinski’s Rule. From Table 2, it was observed that all 6 lead compounds also have percentage human oral absorption more than 50% and allowed logKhsa values. Therefore, it is suggested that good binding ability with human serum albumin and reasonably good oral human absorption may result into better distribution and good absorption of these lead compounds. P_caco_ values suggested that these lead compounds may result into good absorption of these compounds through intestine, which is must for good absorption of a drug through oral route. However, additionally the docking study was performed to predict how the designed potential antimalarial compounds will bind to putative receptor (DHFR). The binding ability of ligands to receptor protein was determined on the basis of glide score found out by molecular docking method performed by method given in Additional file 1.

**Table 2:** The ADME properties and Glide score of the selected six lead candidates

Lead compounds	logPo/W^a^	logS^b^	PCaco^c^	loghsa^d^	logBB	logKp	% human oral absorption^e^	Glide score for inhibition of Pf-DHFR (Kcal/mol)
**13**	2.421	− 5.30	27.88	0.09	− 3.01	− 3.93	54.03	− 6.23
**20**	3.841	− 6.17	551.04	0.58	− 0.72	− 3.15	100.00	− 5.41
**7**	4.614	− 6.39	184.56	0.59	− 2.30	− 2.05	81.56	− 5.25
**15**	3.333	− 6.15	100.16	0.31	− 2.42	− 3.06	69.31	− 4.74
**11**	2.778	− 4.19	619.07	0.05	− 0.97	− 2.81	93.17	− 4.64
**18**	2.409	− 3.75	37.21	0.33	− 0.36	− 7.04	96.13	− 4.25
**Cycloguanil**	–	–	–	–	–	–	–	− **4.17**

^a^logPo/w (− 2.0 to 6.5)

^b^logS (− 6.5 to 0.5)

^c^Pcaco < 25 is very poor and < 500 is great

^d^logKhsa (− 1.5 to 1.5)

^e^% human oral absorption (< 25% is poor and > 80% is high)

Table 2 displayed that all 6 hits have diverse glide scores ranging from − 6.230 to − 4.254 which were higher vis-a-vis that of cycloguanil standard antimalarial drug that works through this pathway. The 2-dimensional interaction maps suggested that in the docking site of DHFR, both hydrophobic interactions and hydrogen bonding were the dominant forces. It is well known through various published works and our own experience that hydrophobic and hydrogen binding interaction play pivotal role in complexation of ligands with proteins [32, 33].

Therefore, from the comparison of compounds selected on the basis of predicted MIC values, docking score and ADME analysis respectively, compound no **11**, **15**, **20** were ruled out of the 6 compounds selected on the basis of predicted *pMIC* values. However, we tried to synthesize all 6 lead compounds, but due to practical problems it was not possible to synthesize all the selected compounds. Compounds **7**, **13** and **18** are the only three compounds which could be synthesized.

**Fig. 1:**
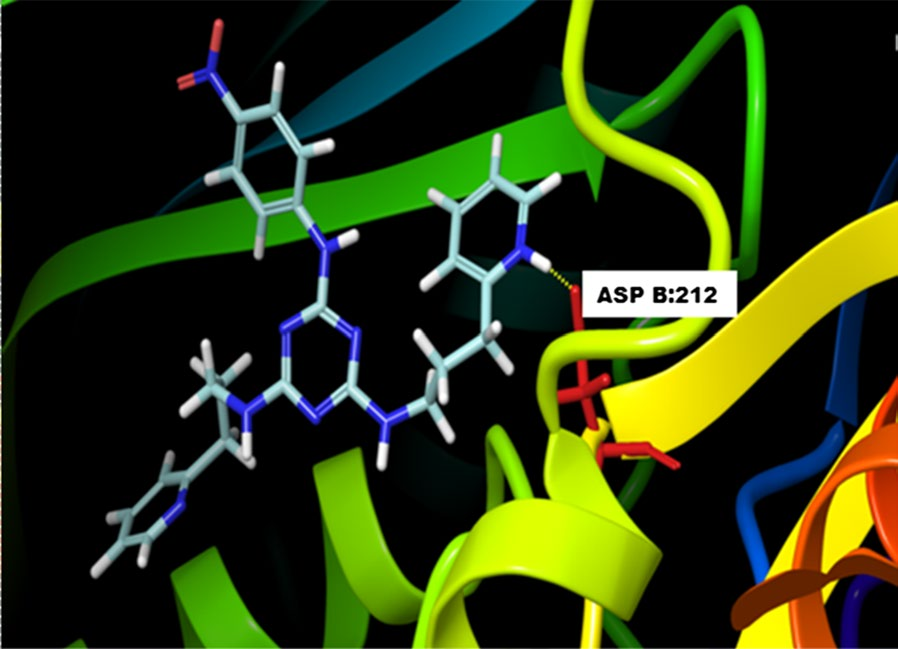
3-D docked for binding of compound no **18** in the active site of DHFR

**Fig. 2:**
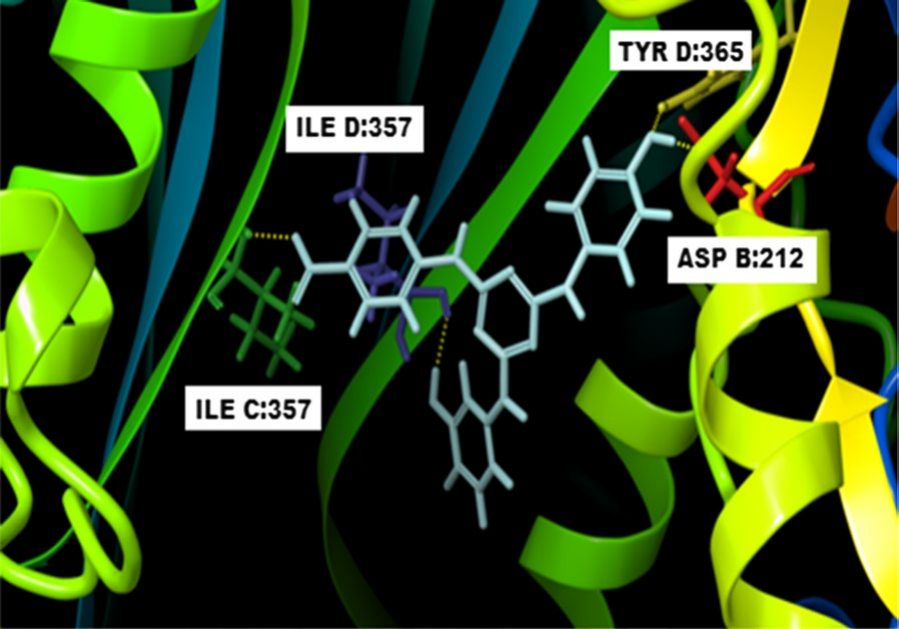
3-D docked for binding of compound no **7** in the active site of DHFR

**Fig. 3:**
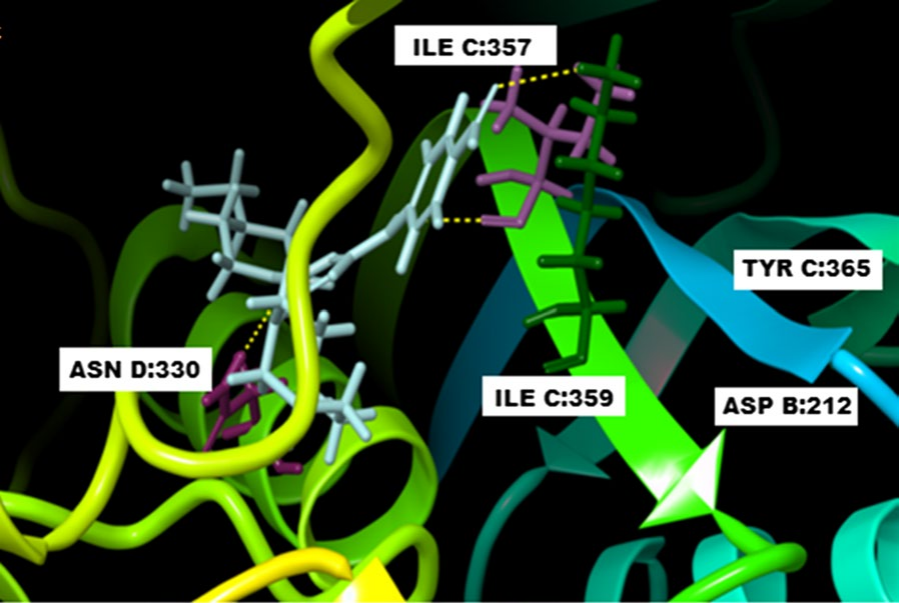
3-D docked for binding of compound no **13** in the active site of DHFR

Figures 1, 2 and 3 showed the 3-dimensional docked models for compounds **18**, **7** and **13** in the binding site of receptor protein DHFR. Compound **18** when docked in the binding site (Fig. 1) formed the hydrogen bond with LYS 359 involving oxygen atom of the nitro group, sandwiched –NH– group with ILE357 and between one of ring nitrogen atoms of triazine ring and ASN330 of the receptor. While the two dimensional interaction map (Additional file 1: Figure S2a) indicated that compound **18** maintained hydrophobic interactions with seven interacting residues in the binding site. Besides, NH^+^ group of piperazine ring formed charged transition with ASP334. Similarly, one of the charged oxygen atom of nitro group formed salt bridge with LYS359.

In compound **7**, there is preponderance of hydrophobic interactions with 11 residues in the binding site (Fig. 2 and Additional file 1: Figure S2b). There was single hydrogen bond formation between nitrogen atom of 2-pyridine and ASP212 of the binding site. Further, on comparing the 2-dimensional interaction maps of compounds **18** and **7**, it was observed that two residues ILE357 and TYR356 of C chain of DHFR were found common in the binding site which formed hydrophobic interactions. Similar to compound **18**, nitro group of the backbone of ligand formed salt bridge with LYS359.

During the docking of compound **13** with *Plasmodium falciparum* DHFR, it was observed that there was formation of four hydrogen bonds between the ligand and four interaction residues of the binding site. One of the phenol group formed hydrogen bond involving oxygen atom ILE357 (D chain) where oxygen atom act as donor and on the contrary, oxygen atom of other substituted phenol ring formed two hydrogen bonds separately with TYR365 and ASP212 respectively and act as hydrogen bond acceptor. The fourth hydrogen bond was formed between O atom of nitro group and residue of chain C ILE357 (Fig. 3 and Additional file 1: Figure S2c). On comparing the two dimensional interaction maps of all three compounds two residues ILE357 and TYR356 were found common for hydrophobic interaction. While there was more than 80% commonness in the interacting residues on the binding sites which play an important role in hydrophobic interactions for compounds **7** and **13**. Therefore, close examination of compounds **7** and **13** suggested that hydrophobic interaction play an equal role for both compounds but compound **13** has more hydrogen bond formation and highest potency.

### Chemistry

**Scheme 1:**
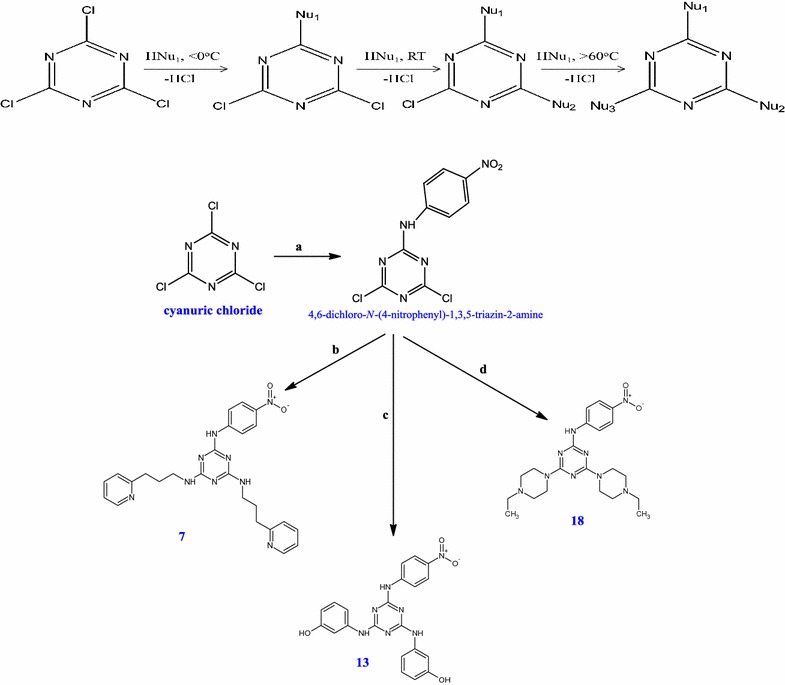
Scheme for the synthesis of 1,3,5-triazine (*s*-triazine) derivatives (**7**, **13** and **18**). Regents: (a) *p*-nitroaniline, K_2_CO_3_ in THF, (b) 1-chloro-4-ethylpiperazine, K_2_CO_3_, 18-Crown-6 in dry THF (refluxed for 2.5 h), (c) 1-chloro-4-ethylpiperazine, K_2_CO_3_, 18-Crown-6 in dry CH_3_CN (refluxed for 3 h), (d) 3-(chloroamino)phenol, K_2_CO_3_, 18-Crown-6 in dry CH_3_CN (refluxed for 3 h)

The compounds **7**, **13** and **18** were synthesized by refluxing 2,4,6-trichloro-1,3,5-triazine (*cyanuric chloride*) with different nucleophiles (R) (Scheme 1). The three compounds were synthesised by some modifications in the literature procedure, which exploits a nucleophilic substitution reaction under solid–liquid phase-transfer conditions. The reaction, which is carried out in the presence of K_2_CO_3_ and a catalytic amount of 18-crown-6, allows possibility to substitute, by employing the appropriate number of equivalents of the nucleophile and K_2_CO_3_, one, two, or all of the chlorine atoms of 2,4,6-trichloro-1,3,5-triazine, only by appropriate reaction temperature. Normally the first substitution in cyanuric chloride takes place at 0 °C, but the amino group of *p*-nitroaniline is highly deactivated due to the presence of a nitro group at the *para* position (Scheme 1 and synthetic scheme in Additional file 1). All the synthesized compounds were characterized by their spectroscopic data, such as IR, NMR, Mass Spectrometry and elemental analysis. The synthesized trisubstituted triazine compounds are shown in Table 3.

**Table 3:** Synthesized trisubstituted triazine compounds

Compound no.	HNu_1_	HNu_2_ = HNu_3_
**7**		
**13**		
**18**		

### Spectroscopic characterization

The skeleton of all three final products (2,4,6-*s*-triazines) have been identified by mass spectrum. The mass spectrum of compound **7** showed a molecular ion peak at *m/z* = 486.6 amu corresponding to (M + H)^+^, which confirms the proposed formula (C_25_H_28_N_9_O_2_)^+^ and its base peak was observed at *m/z* 363.2. Similarly, the molecular ion peak of compound **13** was found at 432.1 and for compound **18** at 442.9 (Additional file 1: Figure S3).

The IR spectra of the compound **7** (Additional file 1: Figure S4) shows a high intensity band at 3412 cm^−1^ due to the presence of intramolecular hydrogen bonding because of the presence of three NH groups. The absorption bands at 1537 and 1372 cm^−1^ are due to NO_2_ stretching vibrations. The IR bands at 1643, 1269, 2841 and 968 cm^−1^ are assigned to C=N, C–N, CH_2_ and N–O stretching vibrations, respectively. The IR peak at 730 cm^−1^ corresponds to a substituted pyridine ring, which further establishes the structure of compound **7**. Similarly, Additional file 1: Figure S5 showed the IR spectrum of the compound **13** displayed the overlapped region of OH and NH stretching bands in the 3200–3400 cm^−1^ region due to the presence of two terminal phenolic groups and three NH groups which are symmetrically attached to the triazine ring. The IR peak at 3409 cm^−1^ corresponds to the O–H stretching vibration. The IR bands at 1631, 1261, 3081 and 1180 cm^−1^ are assigned to C=N, C–N, Ar–H and C-O stretching vibrations, respectively. Also, the presence of the NO_2_ group was confirmed by the presence of two IR stretching bands at 1591 and 1326 cm^−1^.

The IR spectrum of compound **18** (Additional file 1: Figure S6) indicated the absorption band at 3293 cm^−1^ is related to the N–H stretching vibration. The absorption bands at 1559 and 1660 cm^−1^ are due to C=N stretching vibrations, confirming the presence of triazine rings in the compound; the IR bands at 1541 and 1317 cm^−1^ are assigned to NO_2_ stretching vibrations. The presence of the NO_2_ group in the compound is further confirmed by the stretching vibration peak at 966 cm^−1^ corresponding to the N–O stretching vibration. The structure of compound **18** was further confirmed by the presence of absorption bands at 1257, 2845 and 1365 cm^−1^ due to C–N, CH_2_ and tertiary N, respectively.

The ^1^H-NMR spectra have been recorded for compounds **7**, **13** and **18.** In compound **7**, 6 aliphatic methylene groups were found in the region of δ 2.8–4.5 ppm. The 12 aromatic protons were confirmed by the integral of the ^1^H-NMR spectrum and were found to lie in the region of δ 6.3–8.1 ppm. The three NH groups were confirmed by a singlet at δ 5.6 ppm in the ^1^H-NMR spectrum of compound **7** (Additional file 1: Figure S7). The ^1^H-NMR spectra of compound **13** (Additional file 1: Figure S8) confirmed the presence of twelve aromatic protons in the region of δ 7.4–8.9 ppm. The spectrum showed a singlet at δ 9.16 ppm, which confirmed the presence of phenolic protons in the compound. Three different labile protons, belonging to three NH groups, were found as a singlet at δ 5.9 ppm, which further confirmed the structure of compound **13**.

Similarly, the ^1^H-NMR spectra of compound **18 (**Additional file 1: Figure S9), confirms the presence of three chemically non-equivalent methylene proton groups at δ3.9, 3.28 and 3.12 ppm.

The ^1^H-NMR signal at δ 3.5 ppm was caused due to D_2_O exchange and confirms the presence of a labile proton attached to the nitrogen, as the NH group is sandwiched between the two aromatic rings in the compound. The multiplet in the region of δ 7.1–7.6 ppm confirms the presence of four aromatic protons. N terminal aliphatic methyl protons were also confirmed by its deshielded lower δ values.

The ^13^C-NMR spectra was recorded for three compounds; **7**, **13** and **18**. In compound **7**, there were 6 aliphatic methylene groups were found in the region of δ 39.9–64.9 ppm and 19 aromatic carbons in the region of δ 79–172.8 ppm (Additional file 1: Figure S10). The ^13^C-NMR spectrum of compound **13** has showed 21 peaks of aromatic carbons falling in the region of δ 117.7–169 ppm (Additional file 1: Figure S11). In ^13^C-NMR spectrum of compound **18**, 12 aliphatic methylene groups were found in the region of δ 29.5–82 ppm and 9 aromatic carbons in the region of δ 101.4–172.1 ppm (Additional file 1: Figure S12).

### Antimalarial activity evaluation

All the prepared compounds **7**, **13** and **18** were found to be active against the 3D7 strain (cycloguanil sensitive strain) of *Plasmodium falciparum* species and all the analogues show minimum inhibitory concentrations (MIC) in the low nano molar range from 2.75 to 7.94 µmol/L (Table 4).

**Table 4:** MIC values of the synthesized 2,4,6-trisubstituted-1,3,5-triazine derivatives against 3D7 strain of *P. falciparum*

Compound	Minimum inhibitory concentration^a^
**7**	4.466
**13**	7.94
**18**	2.75
**Cycloguanil**	255

^a^Minimum inhibitory concentration for the development of ring stage parasite into the schizont stage during 48 h incubation

The MIC values of the synthesized triazine derivatives were compared with the reported MIC value of cycolguanil, which is taken as a standard drug under the study. The MIC value of cycloguanil has been reported as 64 μg/mL (~ 255 nm) against the NF54 strain of *Plasmodium falciparum* species. Since 3D7 is a drug sensitive laboratory clone of the NF54 isolate and both of them are closely related to each other [34], the reported MIC value of 64 μg/mL was considered reference MIC for comparison with the MIC values of prepared *s-*triazine analogues against the 3D7 strain of the *Plasmodium falciparum* species. It was found, from our activity data of the three synthesized triazine derivatives that all three synthesized compounds were more potent than cycloguanil and compound **18** is the most active out of the three compounds against the *Plasmodium falciparum* species, with an MIC value of 2.75 µmol/L. Compound **7**, with a propyl group linked to 2-pyridine in the substituent part, was found to be less effective against the parasite, than the compound **18**, as its MIC value is 4.466 µmol/L. Compound **13**, was observed to be the least active against the parasite out of the three compounds, as it showed the highest MIC value of 7.94 µmol/L.

This data revealed that the compound **13** with an aromatic ring (with electron donating group) in the substituent part contributed positively towards the antimalarial action. While the ring consisting of electron withdrawing group may cause an increase in the MIC value as showed by compound **7**. Another important feature to correlate biological activity is the number of hydrogen bond formations. In the docking data it was discussed previously that in compound **13** there were four hydrogen bond formations while numbers decreased for compound **18** and least for compound **7**. In the same order, the biological activity varies for the prepared compounds highlighted and established the importance of hydrogen bonding for DHFR inhibition.

Another important dimension in the discussion, which is worth mentioning, is the correlation of two physicochemical properties calculated for these derivatives with MIC values. The first is the LogPo/w values, which describe the lipophilicity of a drug. The calculated LogPo/w values for the above-mentioned compounds **7**, **13** and **18** are 4.61, 2.409 and 2.42, respectively. On matching these with the experimental MIC values of these compounds, it is found that the most potent compound is the least lipophilic one and the trend is that MIC follows those of the lipophilicity. This emphasizes the importance of lipophilicity in the antimalarial activity. The second useful physicochemical property is the coulson charge on the third nitrogen atom placed in the triazine ring of the basic structure of the compounds. On consideration of all five QSAR built models, it is found that out of the six important molecular descriptors, the descriptor which heavily contributes to the activity is the coulson charge of the nitrogen atom in triazine ring, which is sandwiched between two carbons atoms of same ring over which substitution were performed. The property, as obtained computationally, has values − 0.3401, − 0.3555 and − 0.3479, respectively for the three synthesized compounds **7**, **13** and **18**. This property was found to be directly correlated to the MIC value of the compound and this is confirmed by our experimental results. The smallest value of the coulson charge, − 0.3555, was obtained for compound **13** and the least MIC value of 4.2 ± 0.02 nM is also found for the same compound. The other two compounds further confirm to the same trend. This validates the correlation between the structural features of the triazine analogues and their antiplasmodial activity against the *Plasmodium falciparum* species.

## Conclusion

Therefore, in conclusion, the present methodology proved to be a facile and rapid procedure for the preparation of trisubstituted-1,3,5-triazine compounds. The compounds were designed based upon QSAR modelling and further screening was performed on designed inhibitors using ADME and docking studies. An attempt was made to synthesize six compounds, but practically three compounds were synthesized with significantly high yields in the range of 95–100% with high purity, as checked by thin layer chromatography (TLC). The triazine compounds were analysed satisfactorily, both by the spectral and analytical data. The IR, ^1^H-NMR and ^13^C-NMR data have been comprehensively discussed and complement each other for each compound. The triazine derivatives with desired structural features to promote requisite antimalarial property were shown to display desirable antimalarial activity and it was also confirmed using in silico investigations of synthesized inhibitors that hydrogen bonding, lipophilicity and coulson charge were found to correlate with the MIC values. The correlation was performed just to analyze the role of some important pharmacophoric features in DHFR inhibition.

## Additional file

**Additional file 1.** Additional figures.
