# Supplementary material for: Design, synthesis and biological evaluation of antimalarial activity of new derivatives of 2,4,6-s-triazine
Source: Chem Cent J. 2017 Dec 19;11:132. doi: 10.1186/s13065-017-0362-5 (PMC5735044; doi:10.1186/s13065-017-0362-5)
Supplement: Supplementary file 1 — Additional file 1. Additional figures. [file 13065_2017_362_MOESM1_ESM.doc]

**Electronic Supplementary Information**

**Design, synthesis and biological evaluation of antimalarial activity of new derivatives of 2, 4, 6-s-triazine**

Mallika Pathak^1, 2^, Himanshu Ojha^2, 3^*, Anjani K. Tiwari^2^, Deepti Sharma^3^, Manisha Saini^1^, Rita Kakkar^2^

*^1^ Department of Chemistry, Miranda House, University of Delhi, Delhi 110007*

*^2^ Department of Chemistry, University of Delhi, Delhi 110007*

*^3^Division of CBRN Defence, Institute of Nuclear Medicine and Allied Sciences, DRDO, Delhi-110054*

***Corresponding author**

Dr Himanshu Ojha

Division of CBRN Defence,

Institute of Nuclear Medicine and Allied Science,

Timarpur, Delhi 110054

Tel +91-1123905186

Fax+91-11-23919509

Email: [himanshu.drdo@gmail.com](mailto:himanshu.drdo@gmail.com)

**Supplementary Information 1:**

**Molecular Docking**

Glide module from Schrödinger Mastero 9.7. Glide (Grid-based ligand docking with energetic) was used to perform molecular docking. More specifically the docking was performed to screen potential inhibitors based on binding mode and affinity for Plasmodium falciparum DHFR receptor.

The three dimensional target receptor Plasmodium falciparum DHFR (PDB ID: 1J3I) structure was obtained from the protein data bank ([www.rcsb.org](http://www.rcsb.org/)). However, many of these crystal structures or PDBs lack much of the information needed to carry out a rigorous, all-atom simulation of target-ligand interactions. In order to rectify the deficiencies in the structure of protein, for example missing of loop, missing of side chains, etc., a molecular modelling tool called protein preparation wizard in the software has used to obtain optimized protein structure. This step adds missing hydrogen atoms, build in missing residues and loops, identify overlapping atoms, automatically assign missing bond orders, calculate ligand protonation states, and optimize the hydrogen-bonding network

The step also removed unneeded water and hetero molecules beyond 5 Å of the active site in order to save computational cost. In the last step of protein structure preparation, the protein structure was further refined/minimized using a restrained molecular mechanics calculation with the OPLS_2005 force-field to remove potential steric clashes. The structure was minimized until the average root mean square deviation (RMSD) of the non-hydrogen atoms reached 0.3. The quality of the refined protein structure was assessed using Ramachandran plot.

The ligands were prepared using Schrödinger software and ligand preparation (LigPrep) protocol was used to assign bond orders and formal charges. Ionization states and tautomeric states were generated using same protocol. Finally, ligands were energy minimized using OPLS_2005 force field with default settings ensuring the generation of various conformers for the ligands followed by their energy minimization. After preparation of protein and ligand structures suitable for docking calculation, receptor grid files were generated using receptor grid generation program. Under this step, 3D box merely delimits the space where grid points are located and the grid points do not cause receptor atoms to be excluded from the calculation. Without grid, the docking may perform continuum scoring function but the grid will make the docking more time efficient. The potential for non-polar parts of the receptor was softened by scaling down the van der Waals radii of receptor atoms by 1.00 with a partial atomic charge of 0.25. Glide provides three different level of docking precision i.e. high-throughput virtual screening (HTVS), standard precision (SP), extra precision (XP). In the present study, highest precision XP mode of docking program was selected for docking of different ligands with targeted protein [Halgren *et al* 2004, Krovat *et al* 2005].

**References**

Halgren TA, Murphy RB, Friesner RA, Beard HS, Frye LL, Pollard WT, Banks JL(2004)Glide: A new approach for rapid accurate docking and scoring. 2. Enrichment factors in database screening. J. Med. Chem. 47: 1750-1759

Krovat EM, Steindl T, Langer T(2005) Recent advances in docking and scoring. CurrComput. Aided Drug Des. 1: 93-102.


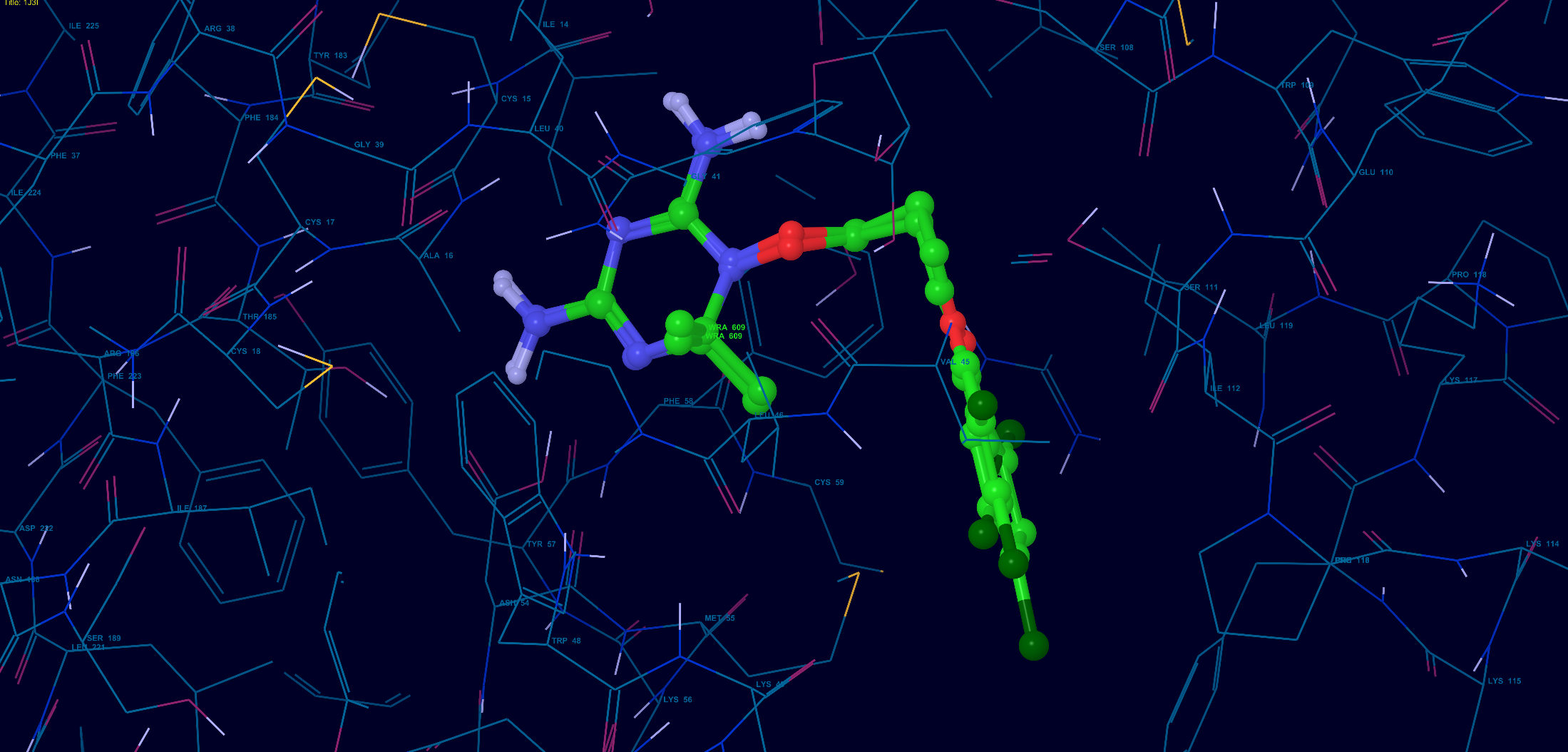


**Fig. S1** The comparison of the structure of WR99210 in the crystal structure of Plasmodium falciparum dihydrofolate reductase-thymidylate synthase (PDB ID: 1J3I) with the structure of WR99210 in the docked Plasmodium falciparum dihydrofolate reductase-thymidylate synthase.


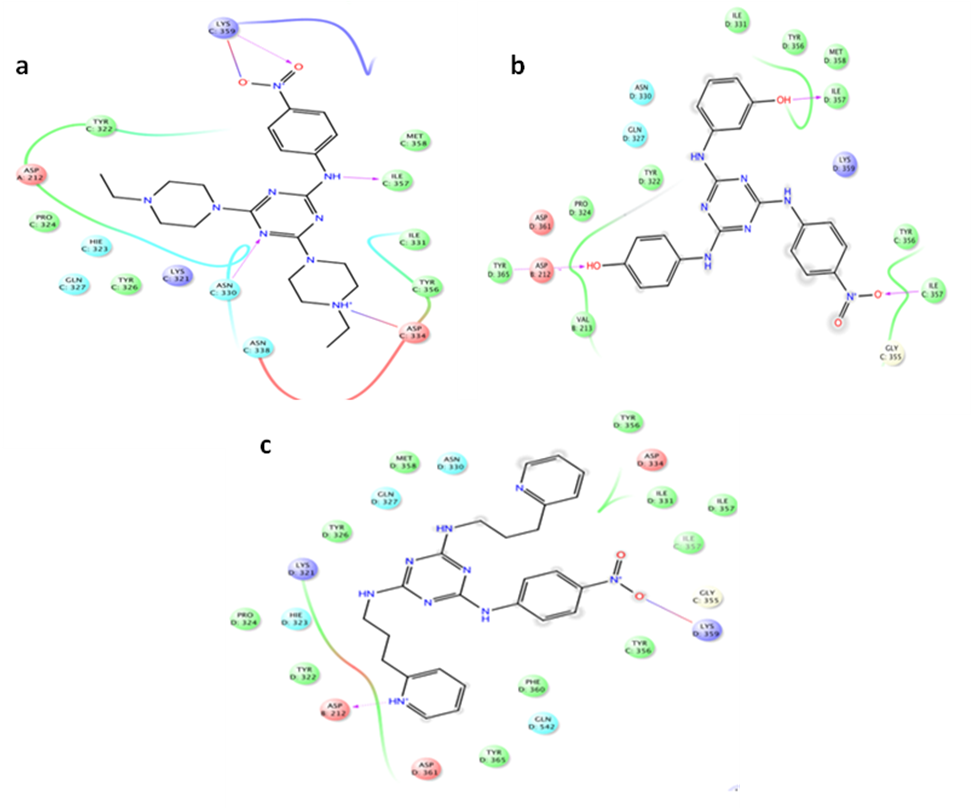


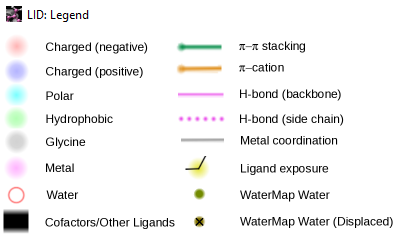


**d**

**Fig. S2** Two dimensional interaction view of binding of compounds no (**a**) **18**, (**b**) **7**, (**c**) **13** at the active site of DHFR; and **(d)** legends.


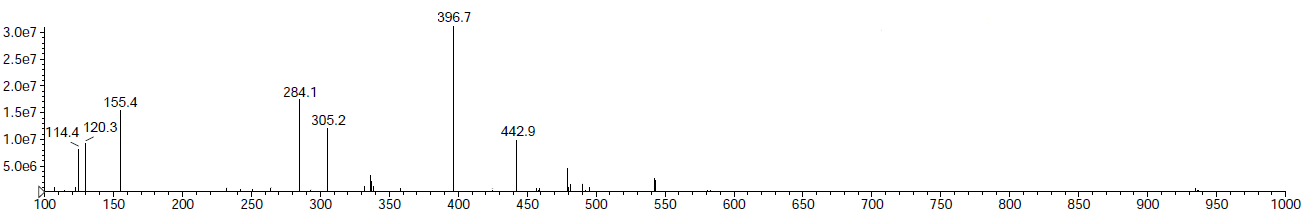


**Fig. S3** Mass Spectra of the compound **18**


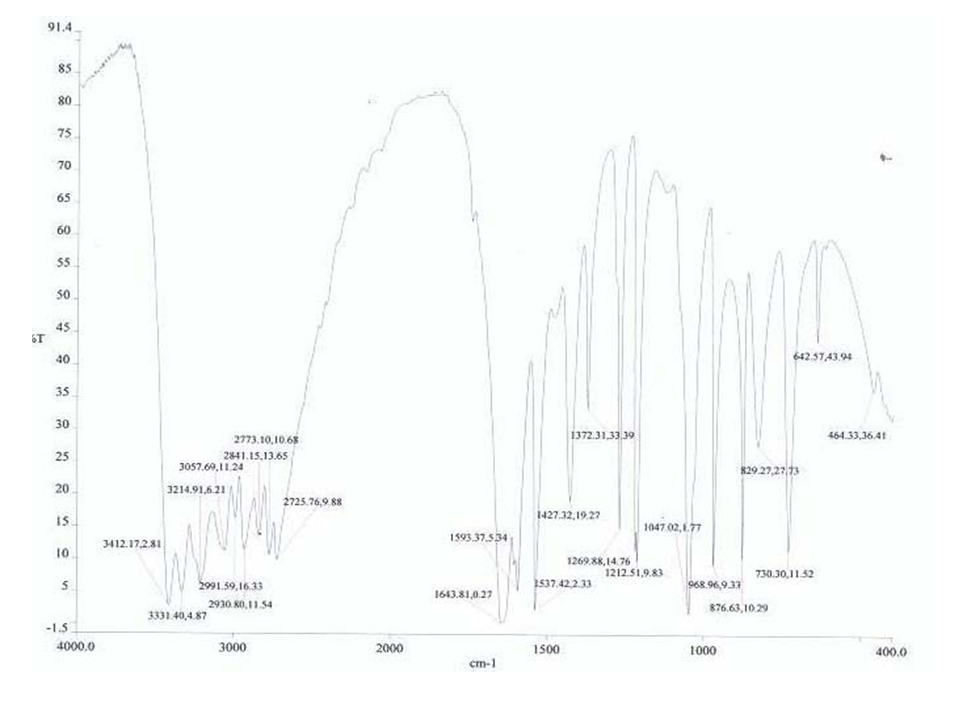


**Fig. S4** IR spectra of compound 7.


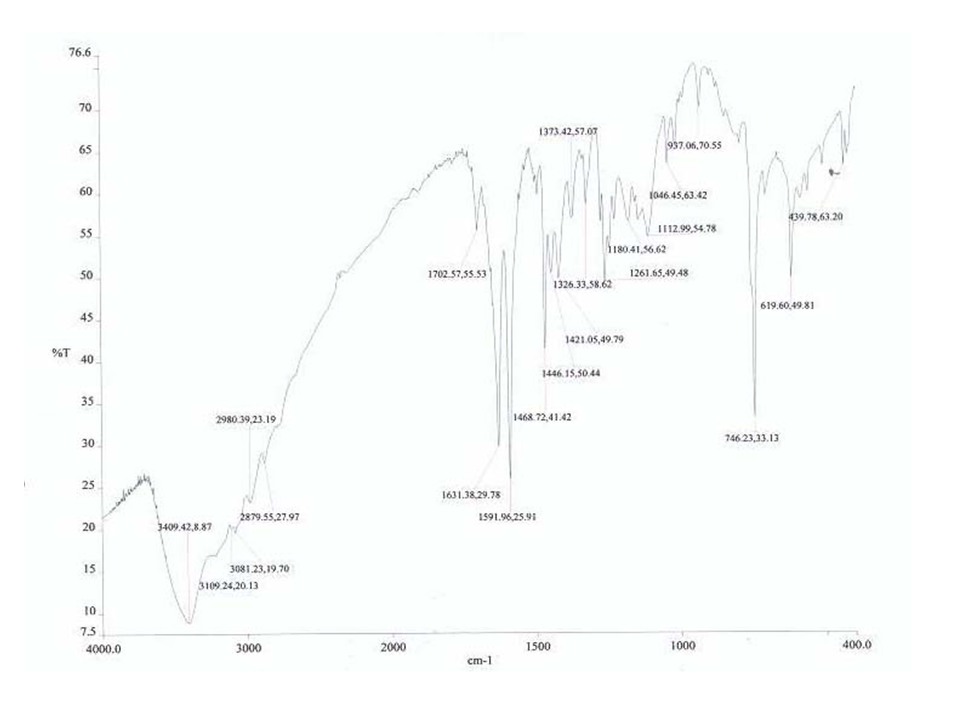


**Fig. S5** IR spectra of compound 13.


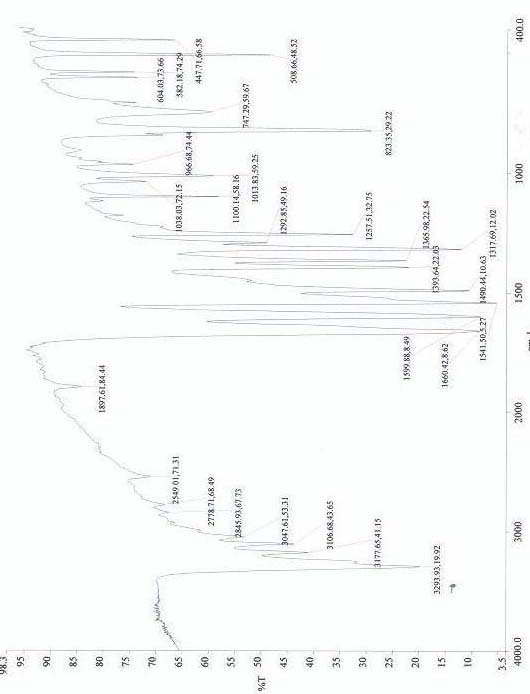


**Fig. S6** IR spectra of compound 18.


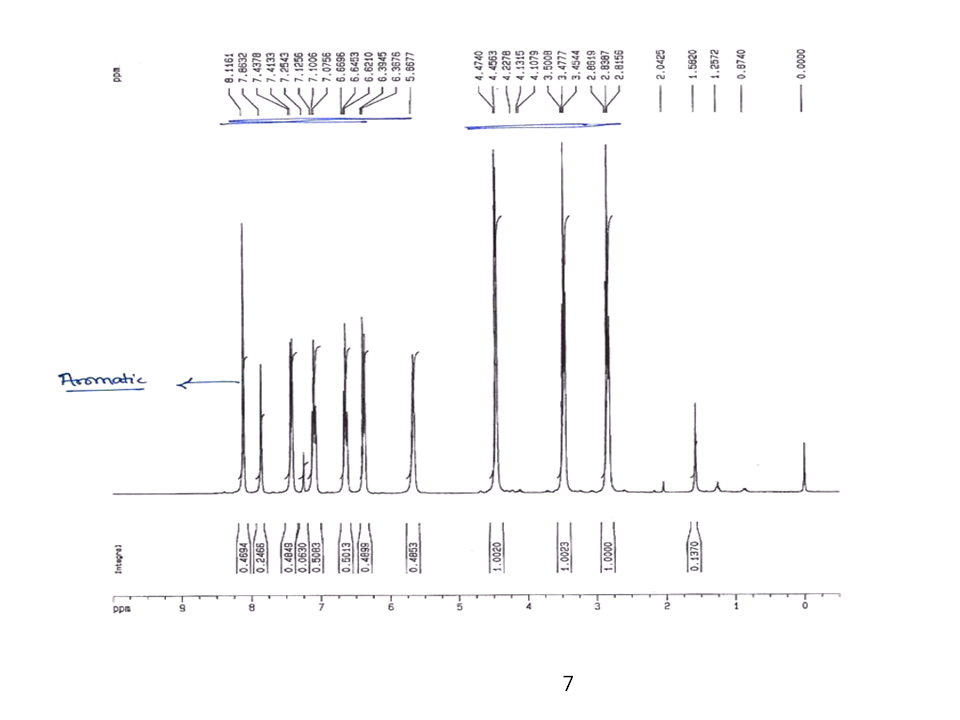


**Fig. S7** ^1^HNMR spectrum of compound 7.


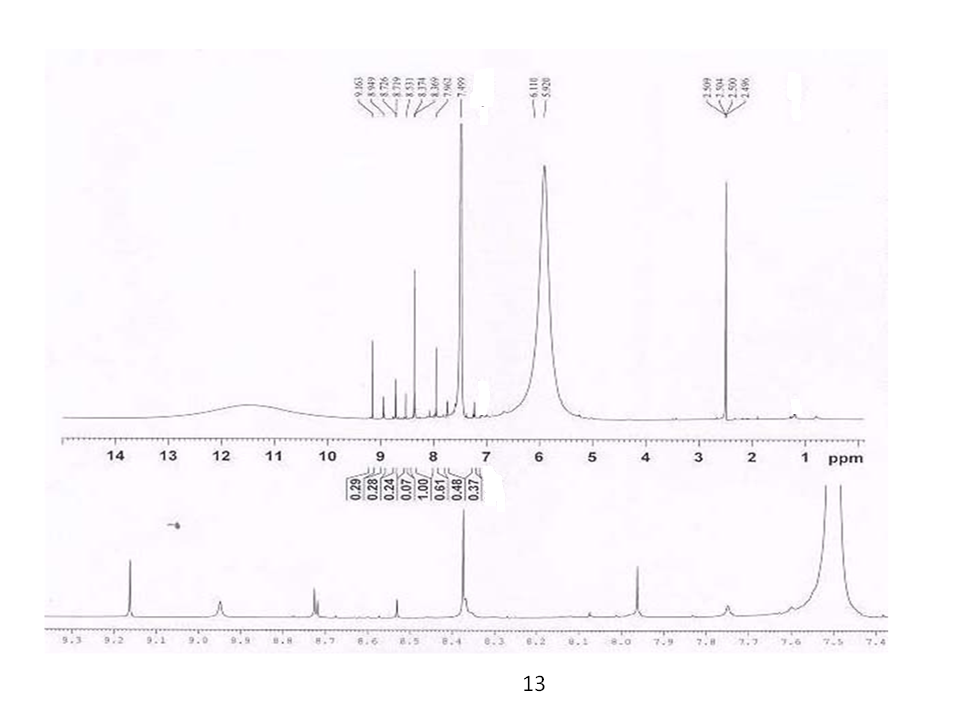


**Fig. S8** ^1^HNMR spectrum of compound 13.


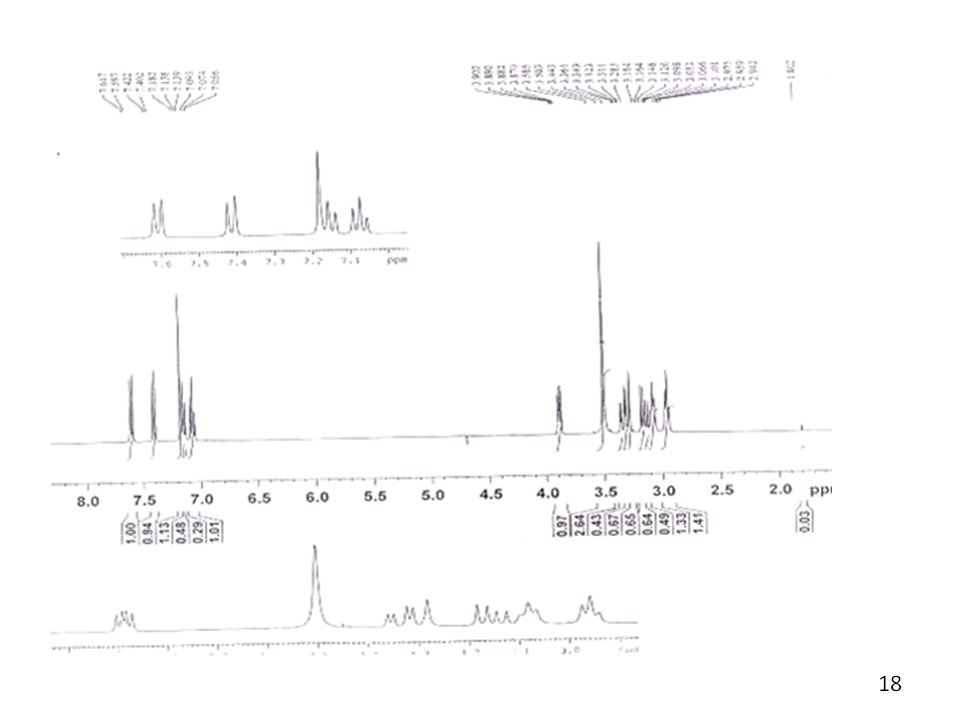


**Fig. S9** ^1^HNMR spectrum of compound **18**

**
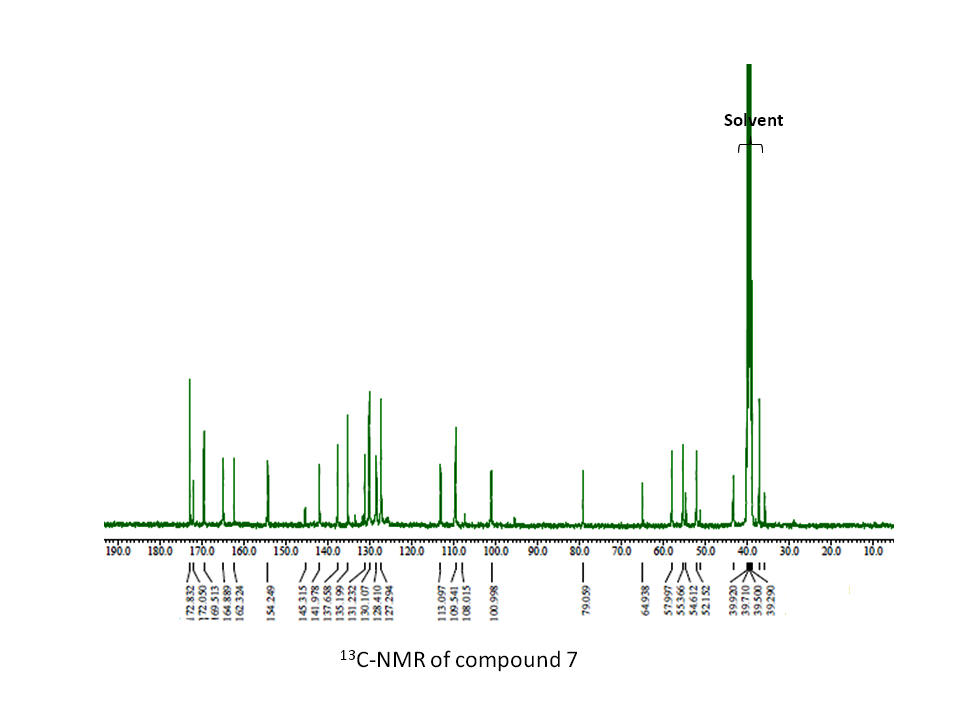
**

**Fig. S10** ^13^C-NMR spectra of compound 7


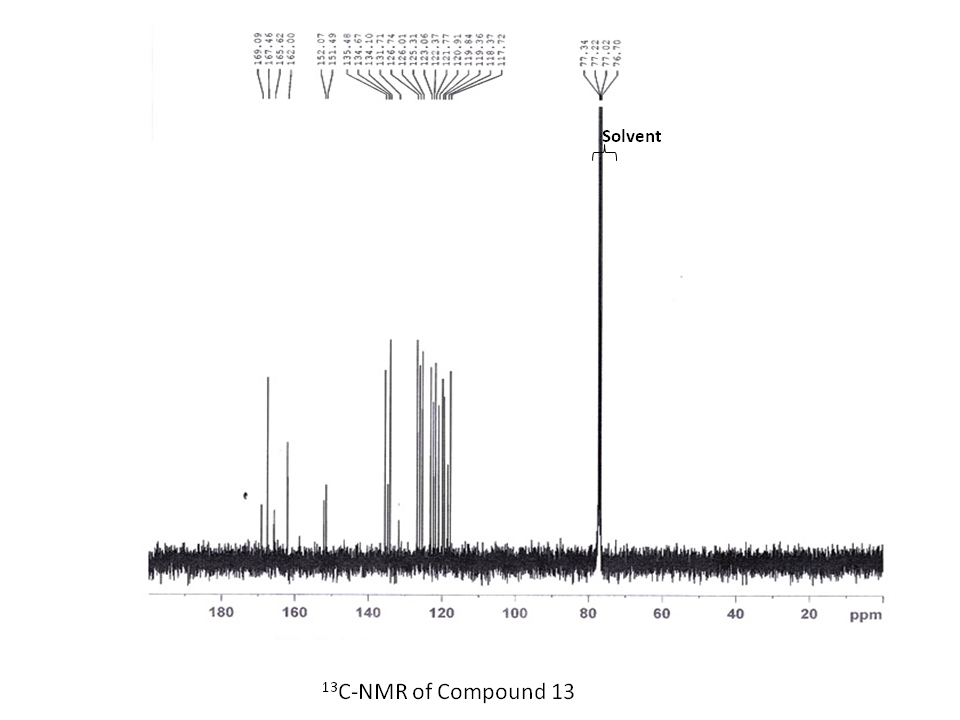


**Fig. S11** ^13^C-NMR spectra of compound 13


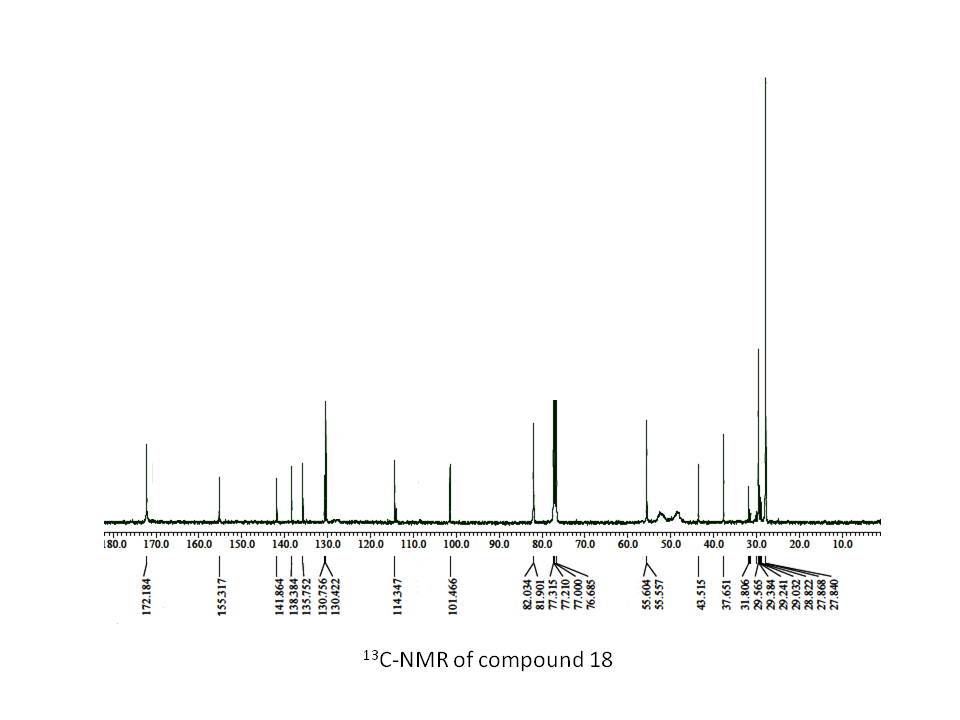


**Fig. S12** ^13^C-NMR spectra of compound 18

**Supplementary Information 2:**

**Synthetic scheme:**

The 2, 4, 6-trisubstituted-1,3,5-triazine compounds were synthesized from 2,4,6-trichloro-1,3,5-triazine (*cyanuric* *chloride*) by reacting it with different nucleophiles. The mono-substituted triazine (4,6-dichloro-N-(4-nitrophenyl)-1,3,5-triazin-2-amine) was synthe­sized by refluxing *cyanuric chloride* with *p*-nitroaniline in the presence of potassium carbonate in tetrahydrofuran (THF).

**4,6-bis(4-ethylpiperazin-1-*yl)-N*-(4-nitrophenyl)-1,3,5-triazin-2-amine (7)** was synthesized by mixing 2.75 mmol of mono-substituted *cyanuric chloride* (4,6-dichloro-N-(4-nitrophenyl)-1,3,5-triazin-2-amine) in dry THF (25 ml) with corresponding 5.50 mmol of the nucleophile 1-chloro-4-ethylpiperazine in the presence of 5.50 mmol K_2_CO_3_ and 18-Crown-6 as a catalyst. The mixture was then refluxed for 2.5 hrs. The mixture was filtered and the filtrate was poured into crushed ice. The crude product obtained was recrystallized from absolute alcohol.

***N2*-(4-nitrophenyl)-*N4,N6*-bis(3-(pyridin-2-yl)propyl)-1,3,5-triazine-2,4,6-triamine (13)** was obtained as the only product when 2 mmol of mono-substituted *cyanuric chloride* (4,6-dichloro-N-(4-nitrophenyl)-1,3,5-triazin-2-amine) in dry CH_3_CN (30 ml) were mixed with 6.0 mmol of K_2_CO_3_ and 6.0 mmol of *N*-chloro-3-(pyridin-2-yl)propan-1-amine as a nucleophile in the presence of 18-Crown-6 as a catalyst. The reaction mixture was refluxed for 3 hrs. The mixture was filtered and the filtrate was poured into crushed ice. The crude product obtained was recrystallised from absolute alcohol.

**3,3'-({6-[(4-nitrophenyl)amino]-1,3,5-triazine-2,4-diyl}diimino)diphenol (18)** was synthesized by mixing 2.0 mmol of mono substituted *cyanuric chloride* (4,6-dichloro-N-(4-nitrophenyl)-1,3,5-triazin-2-amine) in dry CH_3_CN (30 ml) with corresponding 6.0 mmol of nucleophile 3-(chloroamino)phenol in the presence of 6.0 mmol K_2_CO_3_ and 18-Crown-6 as a catalyst. The mixture was then refluxed for 3 hrs. The mixture was filtered and the filtrate was poured into crushed ice. The crude product obtained was recrystallized from absolute alcohol.

**Table S1. Obtained quantitative structure activity relationships models for the molecules studied**

|  |  | Statistical Characteristics | | | |
| --- | --- | --- | --- | --- | --- |
| S.No. | Equations | Lack of Fit | R^2^ | R^2^_CV_ | F-value^a^ |
| 1 | -0.9591^3^χ – 0.0006338E_T_ – 0.09597µ_X_ -42.19q­_N_ – 18.00 | 0.5564 | 0.7722 | 0.5460 | 11.86 |
| 2 | 0.1211^1^κ – 0.9498^3^χ -0.1028µ- 34.34q_N_ – 14.83 | 0.5624 | 0.7697 | 0.6469 | 11.70 |
| 3 | -0.2503^3^SC - 0.0009776E_T_ - 0.001776O_yyy_ -40.66q_N_ – 18.10 | 0.5674 | 0.7677 | 0.6160 | 11.57 |
| 4 | 0.1210^1^κ – 0.9482^3^χ - 0.1029µ_x_ - 34.07q_N_ - 14.67 | 0.5712 | 0.7661 | 0.6399 | 11.47 |
| 5 | 0.1247^1^κ -0.9538^3^χ - 0.1036µ_x_ - 33.46q'_N_ -15.88 | 0.5674 | 0.7595 | 0.6317 | 11.05 |

^a^Critical SOR F-value (95%) = 3.16.

**Ref:** Ojha H, Gahlot P, Tiwari AK, Pathak M, Kakkar R(2011) Quantitative structure activity relationship study of 2,4,6-trisubstituted-s-triazine derivatives as antimalarial inhibitors of *Plasmodium falciparum* dihydrofolate reductase. Chem. Biol. Drug Des.77: 57-62.
